# DP202216 maize hybrids shift upper limit of C and N partitioning to grain

**DOI:** 10.3389/fpls.2025.1459126

**Published:** 2025-03-12

**Authors:** Francisco Palmero, Javier A. Fernandez, Jeffrey E. Habben, Jeffrey R. Schussler, Ben Weers, James Bing, Trevor Hefley, P. V. Vara Prasad, Ignacio A. Ciampitti

**Affiliations:** ^1^ Department of Agronomy, Kansas State University, Manhattan, KS, United States; ^2^ School of Agriculture and Food Sustainability, The University of Queensland, St Lucia, QLD, Australia; ^3^ Research and Development, Corteva Agriscience, Johnston, IA, United States; ^4^ Department of Statistics, Kansas State University, Manhattan, KS, United States; ^5^ Department of Agronomy, Purdue University, West Lafayette, IN, United States

**Keywords:** corn, AG099, NHI, HI, yield stability

## Abstract

Increasing both harvest index (HI) and nitrogen (N) harvest index (NHI) is a promising approach for improving the effective use of resources in grain crops. Previous studies on maize (*Zea mays* L.) reported increments in different carbon (C)-N physiological and morphological traits in DP202216 hybrids (*ZmGos2-zmm28, event DP202216, Corteva Agrisciences*). The objectives of this study were to i) identify changes in the maximum limit (potential) of C and N partitioning to the grains (HI and NHI, respectively) in DP202216 maize hybrids at equal plant growth levels, and ii) determine the main factors underpinning the mechanisms associated with any observed changes in C and N partitioning to grains. Two DP202216 hybrids were evaluated with their respective wild-type (WT) controls during two field growing seasons (2022 and 2023) under three N rates (0 kg ha^-1^, 150 kg ha^-1^, and 300 kg ha^-1^). Long-term ^15^N labeling was used to precisely study N remobilization fluxes. The DP202216 plants showed an increase of 2% and 5% in the upper boundary of the HI and NHI, respectively. Furthermore, the DP202216 hybrids incremented 19% the relative allocation of ^15^N to grains. This was translated into a higher utilization of N absorbed during vegetative stages in DP202216 hybrids, independently of the amount of N uptake. The hybrids with the DP202216 event increased 9% the number of grains per unit of plant biomass. Our study describes improvements on the upper limit of HI and NHI in DP202216 maize hybrids. We showed that the increase in C and N allocation to the reproductive organs in the DP202216 hybrids was related to higher ‘relative’ C and N demand by the grains. Thus, the DP202216 trait provides a new genetic tool to improve grain yield potential and yield stability via enhanced resource utilization in maize production, offering the farmers the opportunity to maximize return on investment (ROI) for N input costs.

## 1 Introduction

Improving resource capture and utilization by crops is crucial to increase crop productivity in a sustainable way (Fischer and Connor, 2018). Increasing the proportion of nitrogen (N) allocated to the grains, i.e. N harvest index (NHI), is a promising approach for improving the effective use of N in grain crops (Lemaire and Ciampitti, 2020). Furthermore, increases in NHI are often associated with an increase in carbon (C) allocation to grains, i.e. harvest index (HI) (Sinclair, 1998; Ciampitti and Lemaire, 2024). Therefore, both NHI and HI should be included as target traits in breeding programs to raise the effective use of resources in grain crops (Ciampitti and Lemaire, 2022).

In maize (*Zea mays* L.), there exists a well-known interdependency among C and N metabolisms during the vegetative period (Gastal et al., 2015). This interrelationship is also present during the reproductive period for both C and N remobilization (Fernandez et al., 2020). Therefore, plant dry matter and N uptake influence the allocation of C and N to grains (Caviglia et al., 2014). Thus, to uncover physiological changes, breeding progress in both HI and NHI traits must be interpreted considering absolute plant dry matter in the analyses because of differences in plant growth observed across a range of biomass (Ciampitti et al., 2022).

Based on the above paragraph, different factors affecting plant growth can impact on C allocation to the grains in maize as it was demonstrated for plant density (Echarte and Andrade, 2003), planting date (Bonelli et al., 2016), and N, phosphorus, and potassium fertilization (Ciampitti and Vyn, 2012; Hütsch and Schubert, 2017). Furthermore, historic maize breeding programs have increased C allocation to grains due to increments in reproductive biomass and higher number of green leaves at physiological maturity (Saenz et al., 2025). Recent maize breeding programs developed short-stature hybrids with higher ability to partition C and N to grains (Kosola et al., 2023). From an N perspective, the N partitioning in crops can be studied using ^15^N-labeling techniques to measure N allocation in different organs. These methods are based on short- or long-term labeling (Gallais et al., 2006).

In maize, when ^15^N fertilizer is applied before rapid vegetative growth, leaves and stems are labeled, while grains are labeled only because of N remobilization (Gallais et al., 2006). Therefore, the application of ^15^N early in the plant cycle allows the determination of N remobilization from the stem and leaves to grains (Cliquet et al., 1990). That is ^15^N labelling is needed to trace N pathways within crop plants allowing the precise determination of N remobilization fluxes between different organs of the plants. Then, for long-term ^15^N labeling, the calculation of the relative ^15^N-specific allocation (RSA) can be implemented to study the N remobilization fluxes (Gallais et al., 2006).

**Figure 1:**
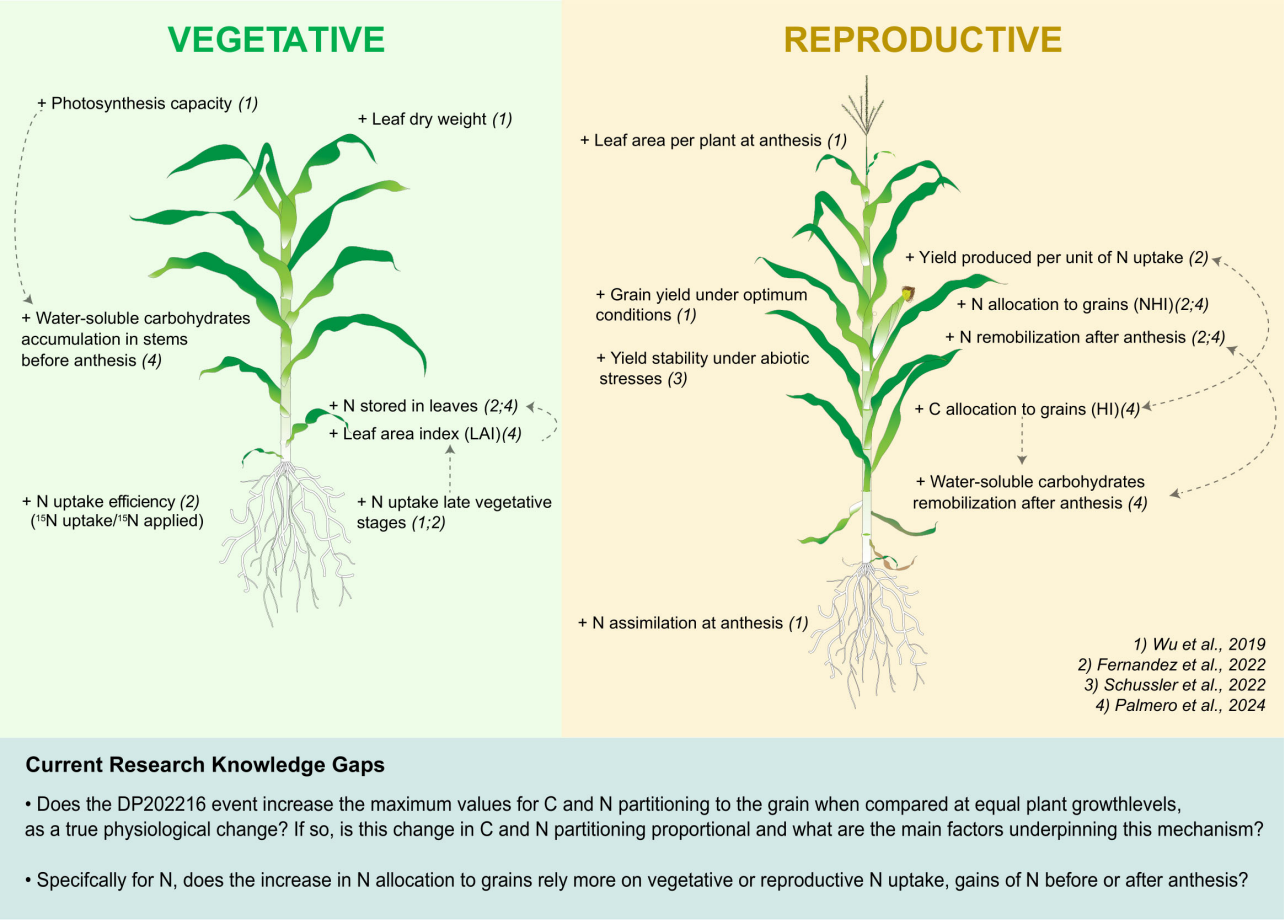
Schematic representation, adapted from Fernandez et al. (2022a), showing the current knowledge of physiological and morphological changes in DP202216 maize hybrids. The plus (+) symbol indicates improved characteristics. Modifications listed in the vegetative panel refer to pre-anthesis changes, while those in the reproductive panel indicate changes at anthesis or post-anthesis. Numbers between parentheses indicate the study in which the change was reported.

Recent studies introduced the impact of the native transcription factor *zmm28* (*ZmGos2-zmm28, event DP202216, Corteva Agrisciences*) on different physiological and morphological traits in maize (Wu et al., 2019; Fernandez et al., 2022a; Schussler et al., 2022; Palmero et al., 2024) (**Figure 1**). Overall, DP202216 hybrids show higher leaf area and photosynthetic capacity, which lead to more water-soluble carbohydrate storage in stems before anthesis. Improved N uptake efficiency leading to a higher N storage in leaves by the end of the vegetative period was observed in DP202216 maize hybrids. This enhanced accumulation of C and N before anthesis subsequently supports more aggressive C and N remobilization to the grain, resulting in higher HI and NHI compared to the wild-type (WT) controls. The enhanced source activity combined with improved remobilization results in more stable grain yield under key abiotic stress conditions, i.e. more yield stability (**Figure 1**).

However, previous studies have not determined the maximum C and N that is allocated to reproductive organs at similar plant growth levels in DP202216 maize hybrids. Doing so, allows for an estimation of the interdependency between plant growth and the N use efficiency (Lemaire and Ciampitti, 2020). Also unknown are the main factors underpinning those possible changes in C and N partitioning. The approach proposed by Milroy et al. (2019) using the frontier analysis for investigating the relationship between plant traits is applied in this article. The frontier analysis indicates, for a given value of plant biomass what is the maximum C and N allocation to the grains that the DP202216 or the WT can achieve. Additionally, we have implemented long-term ^15^N-labeling to separate newly assimilated N from the N taken up earlier in the plant cycle, allowing a precise study of the N remobilization process (Deleens et al., 1994). The objectives of this study were to: i) identify changes in the maximum limit (potential) of C and N partitioning to the grains (HI and NHI, respectively) derived from DP202216 maize hybrids at equal plant growth levels, compared to their WT isogenic hybrids, and ii) determine the main factors underpinning the mechanisms associated with any observed changes in C and N partitioning to grains.

## 2 Materials and methods

### 2.1 Experimental design and crop management

To evaluate the changes in the upper limit of C and N partitioning in DP202216 maize hybrids, two field experiments were conducted in Wamego, Kansas, USA (39° 13’ N, 96° 17’ W), during 2022 and 2023 growing seasons. The soil was a fine, smectitic, mesic Aquertic Hapludolls (Soil Survey Staff, 2022). Chemical soil properties were analyzed before planting each year (**Supplementary Table 1**). In 2022, the experiment was conducted under rainfed conditions. In 2023 a pivot sprinkler system was utilized to irrigate with 19 mm and 32 mm at ten (07/30/2023) and thirty (08/20/2023) days after anthesis (Ritchie and Hanway, 1982), respectively (partial irrigation). Weather information is presented in **Supplementary Table 2**.

Soybean (*Glycine max* (L.) Merr) was the previous crop in both years of the experiment. The sowing dates were 7 June (2022) and 22 May (2023), with a target plant population of 7.8 plants m^-2^ in both growing seasons. The experimental design structure was a split-plot in a randomized complete block with five repetitions. Three N rates (0 kg ha^-1^, 150 kg ha^-1^, and 300 kg ha^-1^) and two maize hybrids (Corteva Agriscience hybrids PH1V69/PH26V11 and PH11V8/PH4HRJ2) were randomized at the main-plot level, and the trait status (DP202216 and WT) was randomized at the sub-plot level. See Wu et al. (2019) for further details of the transformation and backcrossing procedures implemented to obtain the DP202216 and the isogenic WT hybrids.

Conventional tillage (field cultivator with rolling baskets) was used to prepare the seedbed prior to planting with an Almaco (Almaco, Nevada, IA) precision research air planter. Each experimental unit was an eight-row plot with a size of 9.5 m long and 6 m wide. All the N corresponding to each fertilizer N rates was applied fifteen days after planting using 28% liquid urea ammonia nitrate (UAN) as the fertilizer N source. The N was applied as side dressing and injected into the soil through a fertilizer knife attached to a coulter to a depth of 5 cm. Standard pre-emerge and post-emerge (V5) herbicides were used to control broadleaf and grass weed species in the trials. The hybrids all contained one or more insect protection traits, either TC1507-Herculex^®^, or MON810-YieldGard^®^ and MIR604-Agrisure^®^RW, to eliminate potential confounding factors due to insect pressure within locations. Foliar disease development was monitored and found to be below threshold levels, and no chemical fungicides were applied in either year.

### 2.2 Long-term ^15^N isotopic labeling

The ^15^N-labeled fertilizer was applied to plants at V2 stage (Ritchie and Hanway, 1982) to minimize the uptake of ^15^N label after anthesis and promote its distribution in vegetative organs (stem and leaves) (Gallais et al., 2007). The interval between ^15^N-labeled fertilizer application and anthesis was 40 and 42 days in 2022 and 2023, respectively. The ^15^N isotopic labelling was done following the protocol employed in Fernandez et al. (2022b). Briefly, the labeled fertilizer Ca(^15^NO_3_)_2_ was injected into the soil at the base of five consecutive plants in each experimental unit. The ^15^N concentrations in the fertilizer were 10.15% in 2022, and 98% in 2023. The injected fertilizer volume was adjusted to the ^15^N concentration to apply the same amount of ^15^N per plant in both years. Therefore, each plant received 0.700 g and 0.0725 g of fertilizer in 2022 and 2023, respectively.

### 2.3 Sampling, laboratory analyses, and variable calculations

The three central plants (out of the five) labeled with ^15^N isotope were harvested at physiological maturity (R6; Ritchie and Hanway, 1982). Each plant was split into stem, leaves, cob-husks, and grains. Plant samples were dried at 65°C until constant weight. The number of grains per plant was determined, and the plant fraction samples were weighed and prepared for N analyses. For isotopic analyses, the samples were ground through a 0.10 mm sieve, and 3 mg of tissue were weighed and packed in tin capsules. The total N concentration and the ^15^N atom-excess in the tissue samples were determined by an elemental analyzer (PyroCube – Elementar Americas) coupled to an isotope ratio mass spectrometer (visION, Elementar Americas, Ronkonkoma, NY, US) at the Stable Isotope Mass Spectrometry Laboratory at Kansas State University.

The N content in each plant organ (stem, leaves, cob-husk, grains) was obtained as the product between N concentration and dry weight. The total plant biomass and N content were obtained by adding up the dry weight and N content of each organ. The HI was calculated as $HI=\;\frac{Grain\;biomass\;\left(g\;pl^{-1}\right)}{Total\;biomass\;\left(g\;pl^{-1}\right)}$ , and the NHI was computed as $NHI=\;\frac{N\;content\;in\;grains\;\left(g\;pl^{-1}\right)}{Total\;N\;content\;\left(g\;pl^{-1}\right)}$ .

Finally, as per Gallais et al. (2006), the relative ^15^N-specific allocation (RSA) was calculated to quantify the N remobilization flux as $RSA_{i}=\;\frac{A_{t_{i}}\%\;-A_{0_{i}}\%}{A_{f}\%\;-A_{0}{\;}_{i}\%}$ . In this equation, $A_{t_{i}}\%$ represents the ^15^N abundance percentage in the i^th^ organ, $A_{0}{\;}_{i}\%\;$ represents the natural ^15^N abundance percentage in the i^th^ organ, and $A_{f}\%$ represents the ^15^N abundance of the labeled fertilizer. The RSA for stover (RSA_stover_; stem, leaves, and cob-husk), the whole plant (RSA_whole-plant_; stem, leaves, cob-husk, and grains), and the grains (RSA_grain_) were obtained. Since the $A_{f}\%$ was different in each experimental year, the RSA was normalized to analyze both years together. The normalization was done as $Normalized\;RSA=\;\frac{RSA-minRSA}{maxRSA-minRSA}$ , where RSA  is the calculated RSA in a given year and minRSA and maxRSA are the minimum and maximum of RSA for a given year. Normalization or standardization are useful tools for interpreting coefficient regressions under contrasting values on the model inputs (Gelman, 2008; Oka, 2021).

### 2.4 Statistical analyses

Quantile regression was conducted to study whether the DP202216 event raised the upper limit of C and N allocation to grains (Milroy et al., 2019) compared to the WT hybrids. The quantile regressions allow us to model the relationship between the predictor (*x*) and response (*y*) variables at different quantiles of the data distribution. Hence, quantile regression is useful because it enables deeper inference beyond simple descriptors of a distribution like the expected value (mean) or median (0.50 quantile). Bayesian inference was implemented in this study to perform probabilistic inference (quantify the probability that a scientific finding is credible; Ruberg, 2021), simultaneously account for the uncertainty in the parameter estimation, and easily obtain the credible interval of the predictions.

Different deterministic models were fitted to account for linear and non-linear relationships between response and predictor variables. The models were selected according to the posterior predictive distribution and the Widely applicable Bayesian Information Criterion (WBIC) (Gelman et al., 2014).

In the implemented models it was assumed that the year of the experiment, the N rates, and hybrids do not influence the quantile value of the distribution but affect its variance. Therefore, the models were fitted for the DP202216 and the WT separately, and an intercept only model (expected value) with random intercept was considered for the variance implementing the year of the experiment, the N rates, the hybrids, and the blocks as random effects. The selected fitted models were:


(1) $$y_{ijklr}\sim \;asymmetric\;Laplace\left(q_{\tau_{i}},\;\sigma_{jklr}^{2},\tau \right)$$

(2a) $$q_{\tau_{i}}=\;\beta_{0}x_{i}^{\beta_{1}},$$

(2b) $$q_{\tau_{i}}=\alpha x_{i}\text{, or}$$

(2c) $$q_{\tau_{i}}=\omega ,$$

(3) $$\sigma_{jklr}^{2}=e^{\phi +\psi_{jklr}}.$$

**Figure 2:**
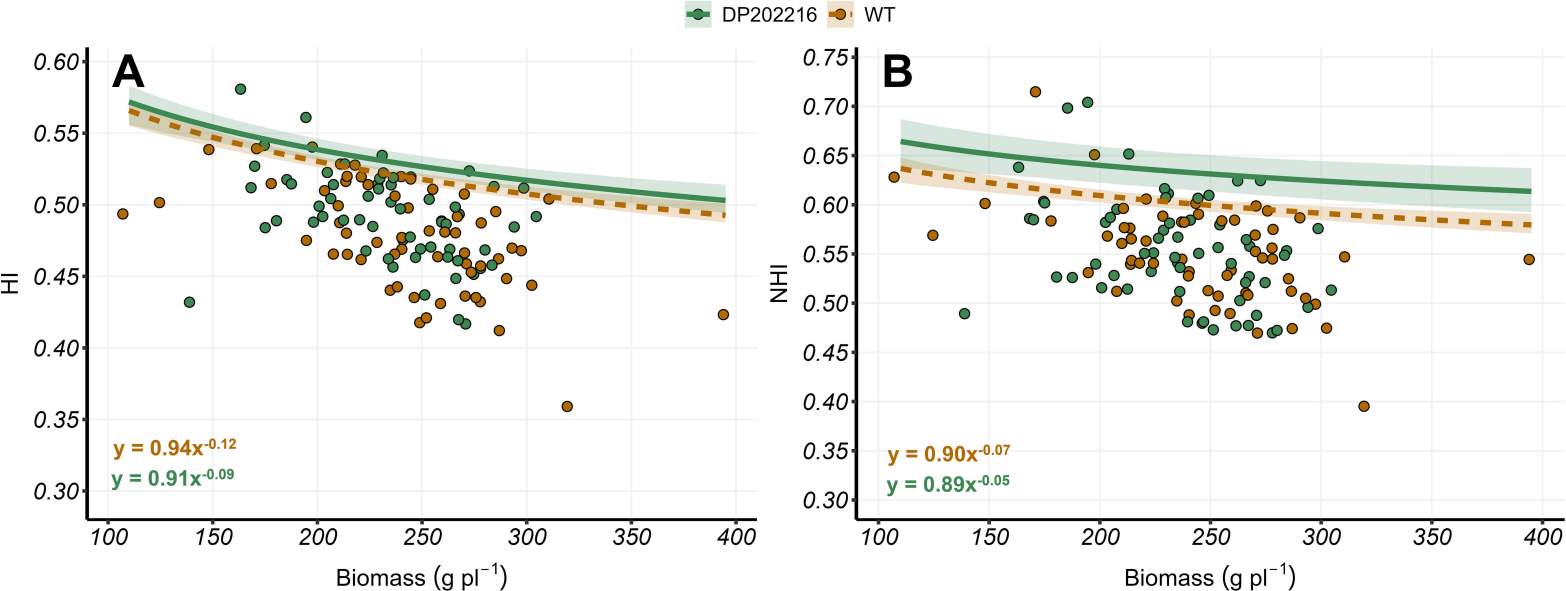
**(A)** Harvest index (HI) versus plant biomass at maturity (R6) and **(B)** nitrogen harvest index (NHI) versus plant biomass at maturity (R6) for DP202216 maize hybrids and their respective wild-type (WT). The solid and dashed lines represent the expected value of the response variable at the 0.95 quantile of the distribution for the DP202216 and the WT, respectively. The shadow area indicates the 95% credible interval of the posterior predictive distribution. The estimated model parameters are: in **(A)** $HI_{i}=0.94x_{i}^{-0.12}$ , for the WT control, and $HI_{i}=0.91x_{i}^{-0.09}$ for DP202216; in **(B)** $NHI_{i}=0.90x_{i}^{-0.07}$ and $NHI_{i}=0.89x_{i}^{-0.05}$ for the WT and DP202216, respectively.

**Figure 3:**
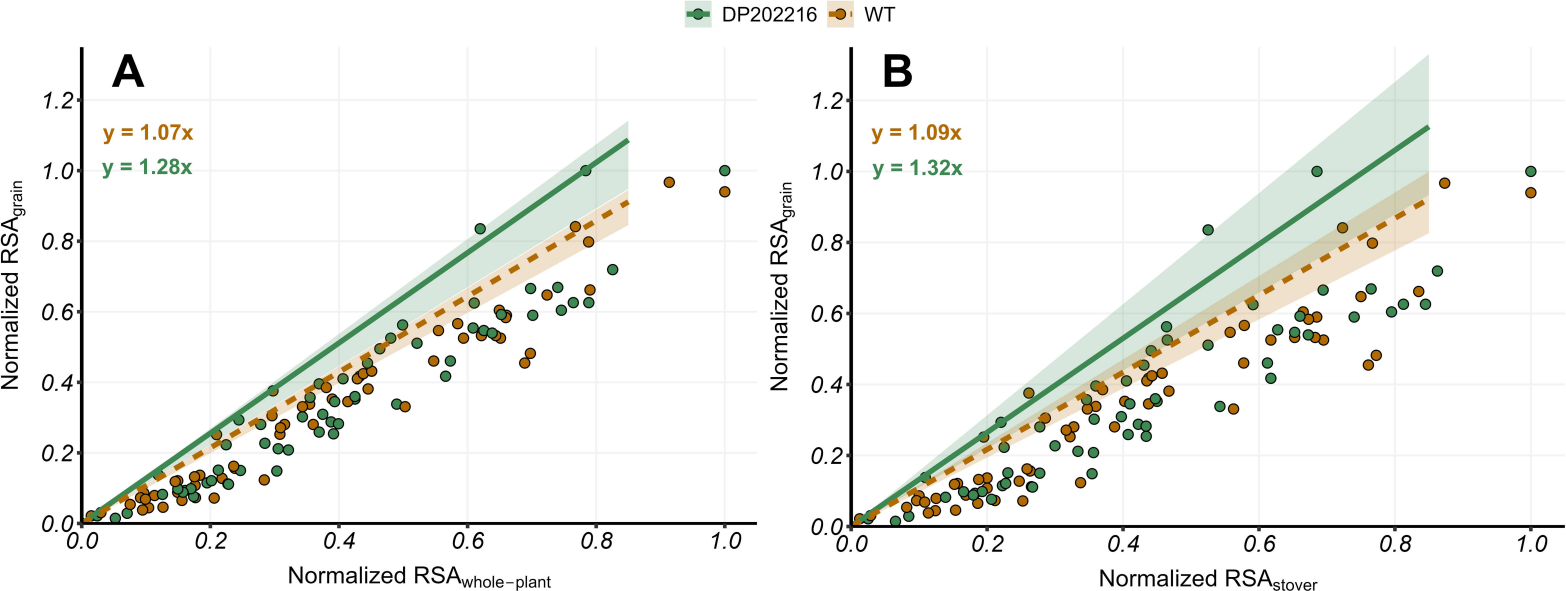
**(A)** Normalized relative ^15^N-specific allocation to grains (RSA_grains_) versus Normalized relative ^15^N-specific allocation to whole-plant (RSA_whole-plant_) at maturity (R6) and **(B)** Normalized relative ^15^N-specific allocation to grains (RSA_grains_) versus Normalized relative ^15^N-specific allocation to stover (RSA_stover_) at maturity (R6) for DP202216 maize hybrids and their respective wild-type (WT). The solid and dashed lines represent the expected value of the response variable at the 0.95 quantile of the distribution for DP202216 maize hybrids and the WT, respectively. The shadow area indicates the 95% credible interval of the posterior predictive distribution. The estimated model parameters are: in **(A)** $RSA_{i,grains}=1.07x_{i}$ for the WT control, and $RSA_{i,grains}=1.28x_{i}$ for DP202216; in **(B)** $RSA_{i,\;grains}=1.09x_{i}$ and $RSA_{i,grains}=1.32x_{i}$ for the WT and DP202216, respectively.

**Figure 4:**
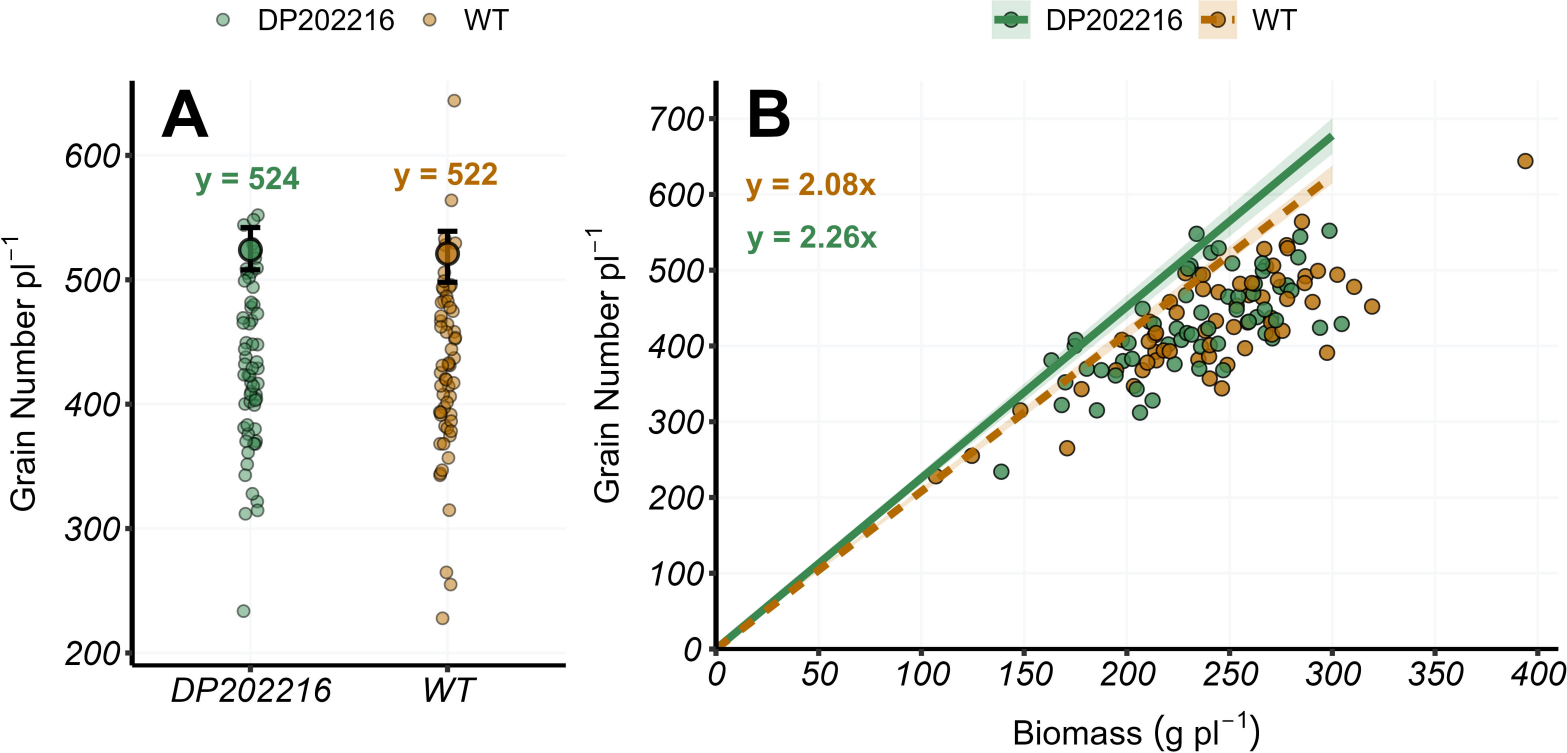
**(A)** Grain number per plant at maturity (R6) and **(B)** grain number per plant versus plant biomass at maturity (R6) for DP202216 maize hybrids and their respective wild-type (WT). In **(A)**, the bigger circles represent the expected value of the grain number per plant at the 0.95 quantile obtained from the posterior distribution of the model parameter (intercept only models), while the whiskers represent the 95% credible interval from the posterior distribution. In **(B)**, the solid and dashed lines represent the expected value of the response variable at the 0.95 quantile of the distribution for the DP202216 maize hybrids and the WT maize hybrids, respectively. The shadow area indicates the 95% credible interval of the posterior predictive distribution. The estimated model parameters are: in **(A)** $Grain\;Number\;pl_{i}^{-1}=522$ , for the WT hybrids control, and $Grain\;Number\;pl_{i}^{-1}=524$ for DP202216 maize hybrids; in **(B)** $Grain\;Number\;pl_{i}^{-1}=2.08x_{i}$ and $Grain\;Number\;pl_{i}^{-1}=2.26x_{i}$ for the WT hybrids and DP202216 maize hybrids, respectively.

Line (1) determines the likelihood function. The subscript *i* is the *i^th^* observation for the DP202216 event or the WT control, *j* represents the *j^th^* year, *k* is the *k^th^* hybrid, *l* represents the *l^th^* N rate, and *r* is the *r^th^* block. The parameter τ is the 0.95 quantile. The Equation 2a-c indicate the deterministic models for the quantile function (instead of the function for the expected value or mean) presented in **Figures 2A, B** (Equation 2a), in **Figures 3A, B**, **4B**; **Supplementary Figure 2** (Equation 2b), and in **Figure 4A**; **Supplementary Figures 1**, **3** (Equation 2c). Furthermore, equation (3) indicates the intercept only model for the variance. The ϕ is the expected value (intercept) of the variance. The symbol $\psi_{jklr}$ represent the random effect of the year of the experiment, the N rates, the hybrids, and the blocks on the variance intercept. An exponential function was applied to ensure only positive values in the variance. Since Bayesian inference was implemented, prior distributions had to be provided for the parameters of each model. See **Supplementary Note 1** for more details about prior selection and hyperparameters.

The probability of difference between model parameters for DP202216 hybrid and its respective WT were computed based on the posterior distribution of the parameters for each model. Only the parameters present in the deterministic part of the model were compared. The probabilities for differences were reported when above 0.65 (weak evidence), 0.75 (moderate evidence), and 0.90 (strong evidence) (Fernandez et al., 2022a; Palmero et al., 2024). For details about programming topics, see **Supplementary Note 1**.

## 3 Results

### 3.1 C and N partitioning

The relationships between HI or NHI versus plant biomass were implemented to study changes on the upper limit of C and N allocation at comparable plant growth levels. In measured DP202216 hybrids, an increase of 2% in the upper boundary of the HI across the studied range of biomass values was observed (**Figure 2A**). This improvement in HI was mimicked by a 5% increase in NHI (**Figure 2B**). Therefore, neither increase nor decrease were observed in grain N concentration relative to the WT controls (**Supplementary Figure 1**). In the fitted model, $q_{{\text{τ}}_{i}}=\;\beta_{0}x_{i}^{\beta_{1}}$ , no differences were found in the $\beta_{0}$ parameters between the DP202216 hybrids and its respective WT controls. However, in the measured DP202216 hybrids, a higher value (closer to zero) of the $\beta_{1}$ parameter was depicted with a probability of difference of 0.69 for the HI-biomass regression (**Figure 2A**) and 0.71 for the NHI-biomass regression (**Figure 2B**). The variability of the response variables (HI and NHI) was not clearly modified by hybrids, N rates, or the year of the experiment (**Supplementary Figure 4**). These results support that lower reduction rate on HI and NHI as plant growth increased (greater $\beta_{1}$ ) may result in DP202216 hybrids showing higher C and N allocation, associated with high levels of plant growth.

### 3.2 Relative ^15^N Specific Allocation

The relationships between RSA_grain_ versus RSA_whole-plant_ and RSA_stover_ were studied to precisely analyze differences in N allocation to the grains between DP202216 and WT control hybrids. In the regressions shown in **Figure 3**, the positive slopes indicate that as *x* was further from the minimum value of *x* (with respect to the range of the explored values in a given year of the experiment), the values of *y* were also further from the minimum value of *y* (with respect to the range of the explored values in a given year of the experiment). Although the response and predictor variables are normalized, the slope of the relationships is still interpreted as changes in *y* per unit of change in *x*.

Under this context, the DP202216 maize hybrids exhibited ^15^N accumulation in grains per unit of ^15^N accumulated in the plant 19% higher than the WT (**Figure 3A**). This pattern was also observed when the changes in ^15^N accumulation in grains were analyzed relative to changes in the ^15^N accumulated in the stover (**Figure 3B**). Thus, DP202216 maize hybrids demonstrated a higher ^15^N accumulation in the grains per unit of ^15^N accumulated in both the plant and the stover with a probability of 0.98 and 0.87, respectively. These differences were consistently observed across both years (**Supplementary Figure 2**), with no significant variation due to hybrids and N rates (**Supplementary Figure 4**). The RSA analyses confirm an improvement in the upper limit of the N allocation to the grains in the DP202216 maize hybrids by separating the N taken up early in the plant growth and development from the N assimilated after anthesis.

### 3.3 Factors underpinning the changes in C and allocation

No differences were found across hybrids in the upper tail of the distribution (0.95 quantile) of the grain number per plant (**Figure 4A**). Consequently, there were no differences in grain biomass per plant (**Supplementary Figure 3**). However, the DP202216 maize hybrids had an increased number of grains per unit of plant biomass (**Figure 4B**). This is represented by the slope of the regression ( $\frac{\Delta y}{\Delta x}=\frac{number\;of\;kernels}{g\;of\;plant\;biomass}$ ), which indicates the number of grains that the WT and DP202216 maize hybrids produced per unit of plant biomass. The slope for the DP202216 maize hybrids was 2.26 while for the WT it was 2.08 with a 0.99 probability of difference for DP202216 maize hybrids >WT hybrids (**Figure 4B**). This difference indicated the DP202216 maize hybrids could maintain, on average, more grains per unit of biomass than the WT controls. The inverse of the slope represented the minimum plant growth needed to maintain one grain, which was 0.44 g grain^-1^ and 0.48 g grain^-1^, for the DP202216 maize hybrids and WT hybrids, respectively. The number of grains grown per plant did not vary across years, hybrids, or N rates (**Supplementary Figure 4**).

## 4 Discussion

This study utilized frontier analyses (0.95 quantile) to demonstrate that the DP202216 maize hybrids showed a higher achievable C and N allocation compared to the WT controls. A comparison using the plant growth achieved at maturity was considered in the analyses revealing changes in the effective utilization of resources (Ciampitti and Lemaire, 2022). The number of grains grown per unit of plant biomass was identified as the main efficiency component driving these changes. We showed an improved N allocation in grain from DP202216 maize hybrids by using long-term ^15^N-labeling, to precisely study N remobilization fluxes in maize (Deleens et al., 1994; Gallais et al., 2007). Furthermore, the frontier analysis proposed by Milroy et al. (2019) was modified by introducing Bayesian statistical inference. This modification allowed us to introduce biological meaning into the models and to make probabilistic inference while accounting for the uncertainty in the parameter estimation.

### 4.1 Modifications on the achievable C and N allocation

DP202216 maize hybrids achieved increased (0.95 quantile) HI and NHI in maize hybrids when compared at the same biomass at maturity. In this study, the HI and NHI were 2% and 5% greater in DP202216 maize hybrid plants with respect to the WT controls, respectively. Palmero et al. (2024) reported increments of ~6% in HI and ~9% in NHI in DP202216 maize hybrids comparing the expected value of the distributions (~0.5 quantile). Our current study depicts DP202216 maize hybrids demonstrated increased upper limit of both C and N remobilization independently of the plant growth, but with a trend of higher differences at high biomass.

The higher HI and NHI observed in DP202216 maize hybrids may be associated to an improved source activity (Wu et al., 2019) leading to higher N and water-soluble carbohydrates accumulation before anthesis with subsequently more aggressive C and N remobilization during the grain filling period (Fernandez et al., 2022a; Palmero et al., 2024). The simultaneous increase in HI and NHI (Lawn, 1989) demonstrates DP202216 maize hybrids may achieve (upper limit) a higher resource use efficiency (Ciampitti and Lemaire, 2024) without compromising the N concentration in grains (Fernandez et al., 2022a). The NHI represents the internal N efficiency, i.e. $\frac{Grain\;N\;}{N\;uptake}$ (Congreves et al., 2021). Therefore, increases in NHI lead to changes in the N use efficiency in grains defined as grain N to N uptake ratio. This higher allocation of N to grains will impact on the grain N concentration depending on the balance between C and N allocation (Sinclair, 1998). We demonstrated that DP202216 maize hybrids have a higher potential N allocation in equilibrium with a higher potential C allocation maintaining grain N concentration (one aspect building seed quality) unchanged.

On the other hand, the N utilization efficiency (yield per unit of N uptake) can be expressed as $\frac{yield}{N\;uptake}=\frac{yield}{Grain\;N}\;\frac{Grain\;N}{N\;uptake}=\;\frac{yield}{Grain\;N}\;NHI$ , showing that increases in NHI lead to increases in N utilization efficiency defined as yield to N uptake ratio. Furthermore, $\frac{yield}{Grain\;N}$ is the inverse of grain N concertation, showing that DP202216 maize hybrids might improve the potential N utilization efficiency via increasing N allocation to the grains (NHI) and not via diluting N in grains (Fernandez et al., 2022a). Therefore, the increase in the potential of C and N allocation observed in DP202216 maize hybrids contributed to maintain grain N concentration without necessarily implying changes in the potential grain number per plant (Fernandez et al., 2022a; Palmero et al., 2024).

### 4.2 N remobilization: long-term ^15^N-labeling

It was shown that DP202216 maize hybrids allocated relatively more N to the grains than the WT maize hybrids at similar levels of relative allocation to plant or stover. These results indicate greater reliance on N absorbed during vegetative stages independently of the amount of N uptake (Fernandez et al., 2022a). Previous research demonstrated higher N remobilization in DP202216 maize hybrids implementing the ‘balance approach’ (Fernandez et al., 2022a; Palmero et al., 2024). The use of long-term ^15^N-labeling allowed us to study the N remobilization flux with higher accuracy by calculating the RSA (Gallais et al., 2006), which does not depend on the amount of N taken up. Therefore, we revealed an improved N allocation to grain in DP202216 hybrids using a more unbiased and robust procedure (Deleens et al., 1994; Gallais et al., 2007).

By applying the ^15^N-labeled fertilizer at V2, the ^15^N uptake after anthesis might have been reduced to only 5% of total seasonal uptake (Gallais et al., 2007). However, the irrigation carried out ten and thirty days after R1 in the 2023 season could increase the ^15^N uptake after anthesis (Gallais et al., 2007). The ^15^N taken up after anthesis may have been incorporated into amino acids in vegetative tissues and then remobilized to the grains due to the protein turnover process (Fernandez et al., 2022c). This could lead to slopes greater than 1 when studying the relationships between RSA_grains_ versus RSA_whole-plant_ or RSA_stover_ (Gallais et al., 2006, Gallais et al., 2007).

### 4.3 Processes underpinning changes on HI and NHI

DP202216 maize hybrids grew more grains per gram of plant biomass than the WT maize hybrids controls. However, no differences were found either in the grain number per plant nor in grain biomass per plant. At a given plant density, the grain number per plant is the main variable explaining changes in maize grain yield (Fernandez et al., 2022b; Ruiz et al., 2022). Two mechanisms via changes in sink strength (sink size and sink activity) may be involved in the observed increased C and N partitioning in DP202216 maize hybrids.

These mechanisms are related to the sink size (grain number m^-2^ or pl^-1^) in ‘absolute’ or ‘relative’ terms and sink activity (rate at which photosynthates are absorbed per unit of grain biomass). When DP202216 maize hybrids accomplish more grain yield than the WT controls (Wu et al., 2019; Schussler et al., 2022), the higher sink size and sink activity may result in an increase in C and N allocation to the reproductive organs (Caviglia et al., 2014). This would imply changes in C and N allocation in ‘absolute’ terms (i.e. depending on the grain number m^-2^ or pl^-1^). When DP202216 maize hybrids show equivalent grain yield with the WT isogenic controls (this study and Fernandez et al., 2022a), DP202216 maize hybrids may grow more grains per unit of plant biomass, thus would promote a higher HI and NHI. This may result in changes in C and N allocation in ‘relative’ terms, expressed in plant biomass.

The suggested mechanisms may result in more effective use of resources for the DP202216 maize hybrids over a wide range of environmental conditions (Ciampitti and Lemaire, 2022). This postulates that when the environmental conditions favor crop growth, the improvements on C and N allocation in DP202216 maize hybrids would result in increments in grain yield (Wu et al., 2019). In contrast, under environmental conditions that do not favor crop growth, the increments on C and N allocation in DP202216 maize hybrids would increase grain yield stability, i.e. lower rate of yield decay under abiotic stress conditions (Schussler et al., 2022; Weers et al., 2024).

## 5 Conclusion

The present study introduces insights into the achievable C and N allocation in DP202216 maize hybrids and the main mechanisms driving these changes. Improvements on the upper limit of HI and NHI, with no changes in grain N concentration, were revealed in DP202216 maize hybrids at comparable plant growth levels. A higher utilization of N absorbed during vegetative stages was reported for DP202216 hybrids, independently of the amount of N taken up. We showed that the increase in C and N allocation to the reproductive organs in DP202216 maize hybrids was related to higher ‘relative’ C and N demand by the grains. Thus, the increased and extended expression of *zmm28* would change the agronomic performance of DP202216 hybrids improving grain yield potential and yield stability in maize hybrids via enhanced utilization of resources. Future research should tackle C and N physiological traits in DP202216 hybrids under abiotic stresses such as drought and limited N conditions to better understand T (trait) x M (management) interactions with yield and yield stability.

## Data Availability

The datasets presented in this article are not readily available because the data that has been used is confidential. Requests to access the datasets should be directed to fpalmero@ksu.edu.
