# Supplementary material for: DP202216 maize hybrids shift upper limit of C and N partitioning to grain
Source: Front Plant Sci. 2025 Mar 12;16:1459126. doi: 10.3389/fpls.2025.1459126 (PMC11937006; doi:10.3389/fpls.2025.1459126)
Supplement: Supplementary file 1 [file DataSheet1.docx]

|  |  | 20 cm depth | | | |  | 60 cm depth | |
| --- | --- | --- | --- | --- | --- | --- | --- | --- |
| Year |  | pH | OM | P | K |  | ${N-N0}_{3}^{-}$ | ${N-NH}_{4}^{+}$ |
|  |  |  | % | mg kg^-1^ | |  | mg kg^-1^ | |
| 2022 |  | 6.1 | 3.54 | 53.4 | 304.0 |  | 27.6 | 6.6 |
| 2023 |  | 5.9 | 3.21 | 59.5 | 301.7 |  | 9.8 | 17.4 |

**Supplementary Table 1**. Chemical soil properties at planting in each of the years of experiment. Values are the average over blocks.

pH was measured in water (1:2.5); OM (organic matter) technique: loss ignition; P (phosphorous) technique: Mehlich-3; K (potassium) technique: ammonium acetate;
${N-N0}_{3}^{-}$ (nitrogen from nitrates) and ${N-NH}_{4}^{+}$ (nitrogen from ammonium) techniques: KCl extraction.

| Year |  | Period |  | T Min  (°C) | T Mean  (°C) | T Max  (°C) | Precipitation  (mm) |
| --- | --- | --- | --- | --- | --- | --- | --- |
| 2022 |  | Emergence-Anthesis |  | 20.1 | 25.7 | 31.4 | 185 |
|  |  | Anthesis-Physiological maturity |  | 17.5 | 24.7 | 31.9 | 154 |
|  |  |  |  |  |  |  |  |
| 2023 |  | Emergence-Anthesis |  | 16.4 | 23.1 | 29.8 | 177 |
|  |  | Anthesis-Physiological maturity |  | 18.0 | 25.8 | 33.7 | 102 |

**Supplementary Table 2**. Weather data for periods emergence-silking and silking-physiological maturity (Ritchie and Hanway, 1982) for each of the years of experiment. The data was retrieved from the closest (< 4 km) National Oceanic and Atmospheric Administration (NOAA) weather station.

T Min (°C): Minimum average air temperature. T Mean (°C): Mean air temperature. T Max (°C): Maximum average air temperature.

**Supplementary Note 1**

The first three parts of the model were presented in the main section of the manuscript. Refer to that section for more details about the likelihood, deterministic models for the 0.95 quantile, and the random intercept model for the variance. Below, we describe the prior distributions implemented for the parameters in each of the fitted models:

$\boldsymbol{\psi}_{jklr}\sim MVN(0, \boldsymbol{\Sigma})$, (3)

$\beta_{0}\sim beta(1,1)$, (4)

$\beta_{1}\sim uniform(-2,0)$, (5)

$\alpha\sim gamma(0.08, 0.04)$, (6)

$\omega\sim gamma(1, 0.002)$, (7a)

$\omega\sim gamma(0.08, 0.04)$, (7b)

$\omega\sim gamma(0.25, 0.005)$, (7b)

$\sigma_{random}^{2} \sim half-student-t(\kappa,\eta, \theta)$, (8)

$\phi\sim half-student-t(\kappa,\eta,\theta)$. (9)

In (3), the random effect for the variance intercept, $\boldsymbol{\psi}_{jklr}$, was assumed to follow a multivariate normal distribution with expected value zero and covariance matrix $\boldsymbol{\Sigma}$**,** where $\boldsymbol{\Sigma}$ is a diagonal matrix with diagonal elements $\sigma_{random}^{2}$. For the deterministic model in (2a), $q_{\tau_{i}}$ is the value of HI or NHI at the 0.95 quantile and the $\beta_{0}$ parameter indicates the value of $q_{\tau_{i}}$ when $x_{i}=1$. Therefore, as shown in (4), it was assumed $\beta_{0}\sim beta(1, 1)$ because $\beta_{0}$can take values only in [0,1]. Furthermore, based on previous experiences, a negative relationship was expected between HI or NHI and biomass. Therefore, in (5) it was assumed that $\beta_{1}\sim uniform(-2,0)$, which provide a wide range of possible values considering the scale of the predictor and response variables. Moreover, a gamma distribution was assumed in (6) for $\alpha$ based on Gallais et al. (2006). The priors in (7a) – (7b) correspond to the only intercept models when grain number, grain N concentration, and grain yield were the response variables, respectively. Hence, gamma distributions were assumed. In (8) and (9), the hyperparameters $\kappa$ and $\eta$, and $\theta$ controls the shape, the location, and the scale of the distribution, respectively. The parametrization of the half-student-t distributions was: $\kappa=3$, and $\eta=0$ for all the models, whereas $\theta$ varied between 2.5 and 68.2 depending on the scale of the response variable in each model.

The posterior distribution of the model parameters was obtained via Hamiltonian Monte Carlo through the No-U-Turn Sampler (an MCMC algorithm). Four chains were run in parallel with 7,000 iterations and warmup period of 3,500. After warmup, one in five draws was saved (i.e. thinning = 5) to reduce autocorrelation among draws before making inference. The convergence of the models was explored via trace plots and Gelman-Rubin diagnostic (Gelman and Rubin, 1992). The models were fitted in R program (R Core Team, 2020, version 4.2.1) via RStudio interface (Posit team, 2023, version 2023.3.0.386), using the Stan programming language through the brms package (Bürkner, 2017).

**
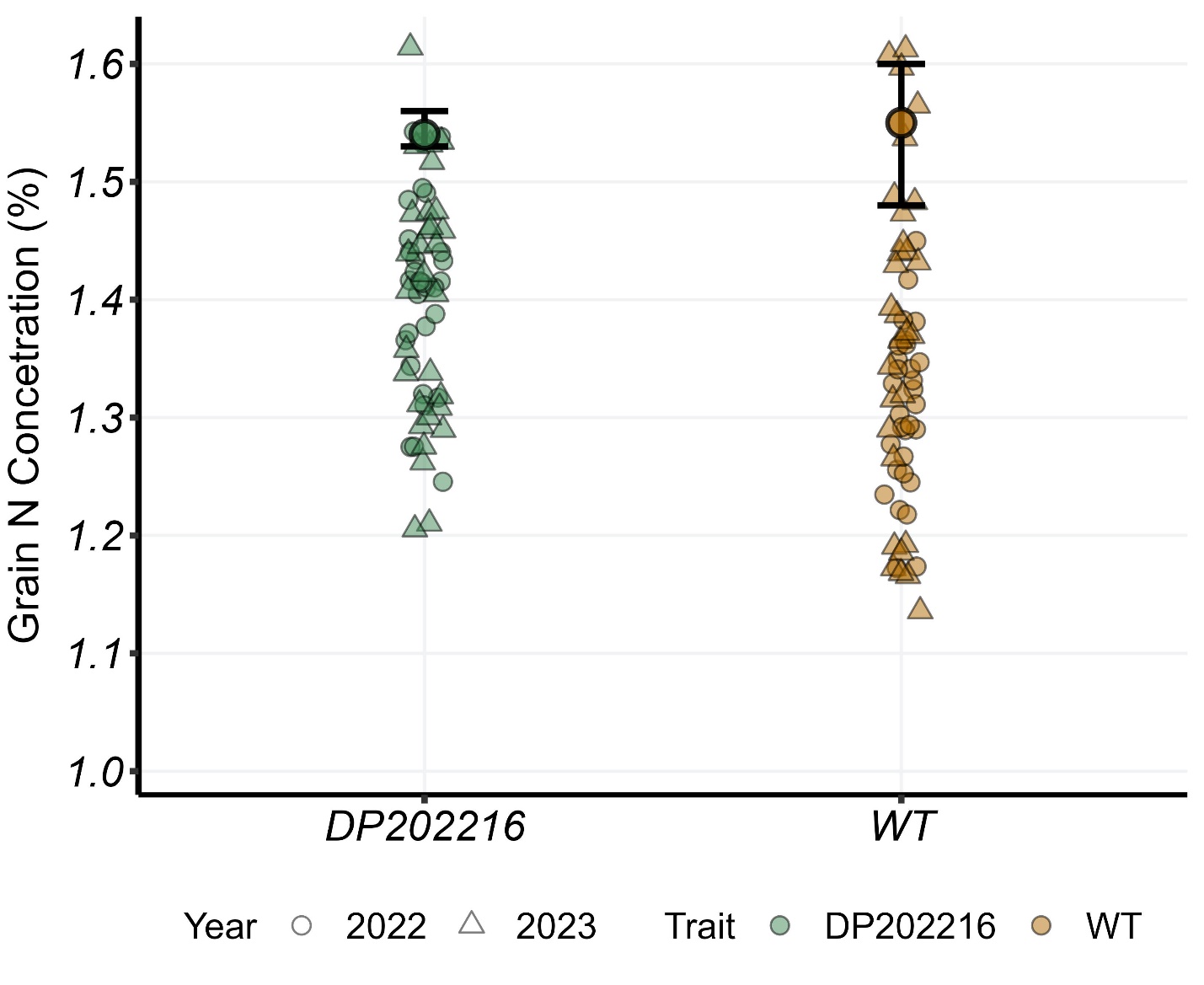
Supplementary Figure 1**. Grain nitrogen concentration for DP202216 corn hybrids and their respective wild-type (WT). The bigger circles represent the expected value of the Grain nitrogen concentration at the 0.95 quantile obtained from the posterior distribution of the model parameter (intercept only models), while the whiskers represent the 95% credible interval from the posterior distribution. The estimated model parameters are: $Grain Concentration \left( \% \right)_{i}=1.55$ for the WT control, and $Grain Concentration \left( \% \right)_{i}=1.54$ for DP202216.

**
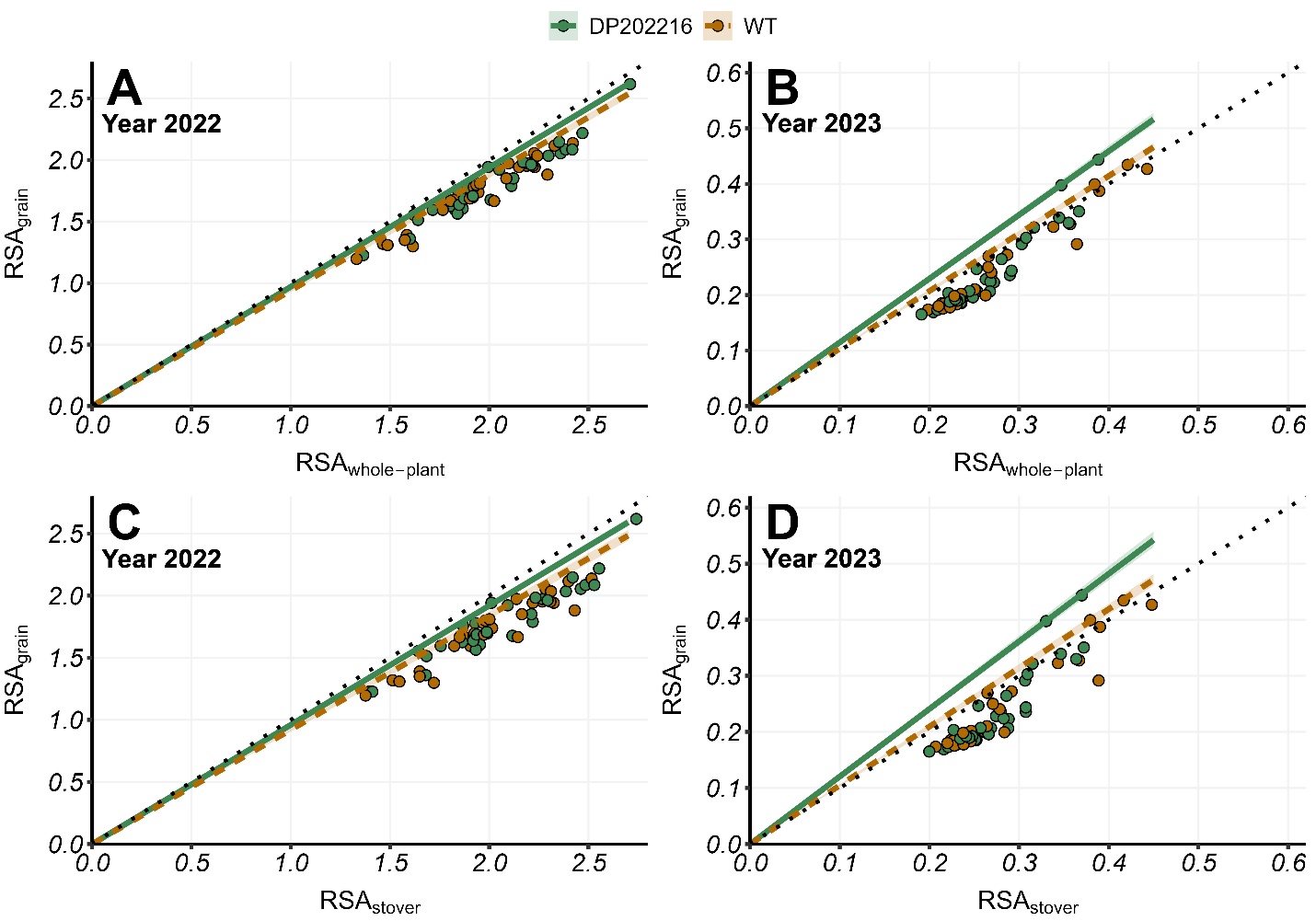
Supplementary Figure 2**. In (A) and (B), relative ^15^N-specific allocation to grains (RSA_grains_) versus relative ^15^N-specific allocation to whole-plant (RSA_whole-plant_) at maturity (R6) in 2022 and in 2023 growing seasons for DP202216 corn hybrids and their respective wild-type (WT). In (C) and (D), relative ^15^N-specific allocation to grains (RSA_grains_) versus relative ^15^N-specific allocation to stover (RSA_stover_) at maturity (R6) in 2022 and 2023 growing seasons for DP202216 corn hybrids and their respective wild-type (WT). The solid and dashed lines represent the expected value of the response variable at the 0.95 quantile of the distribution for the DP202216 and the WT, respectively. The shadow area indicates the 95% credible interval of the posterior predictive distribution. The dotted black line represents a 1:1 relationship. The estimated model parameters are: in (A) $RSA_{i, grains}=0.94x_{i}$, for the WT control, and $RSA_{i,grains}=0.97x_{i}$ for DP202216; in (B) $RSA_{i,grains}=1.04x_{i}$and $RSA_{i,grains}=1.15x_{i}$ for the WT and DP202216, respectively. In (C) $RSA_{i,grains}=0.92x_{i}$, for the WT control, and $RSA_{i,grains}=0.96x_{i}$ for DP202216; in (D) $RSA_{i,grains}=1.05x_{i}$ and $RSA_{i,grains}=1.20x_{i}$ for the WT and DP202216, respectively. In all the panels (A, B, C, and D), the DP202216 event achieved showed a higher slope with probability >0.97.

**
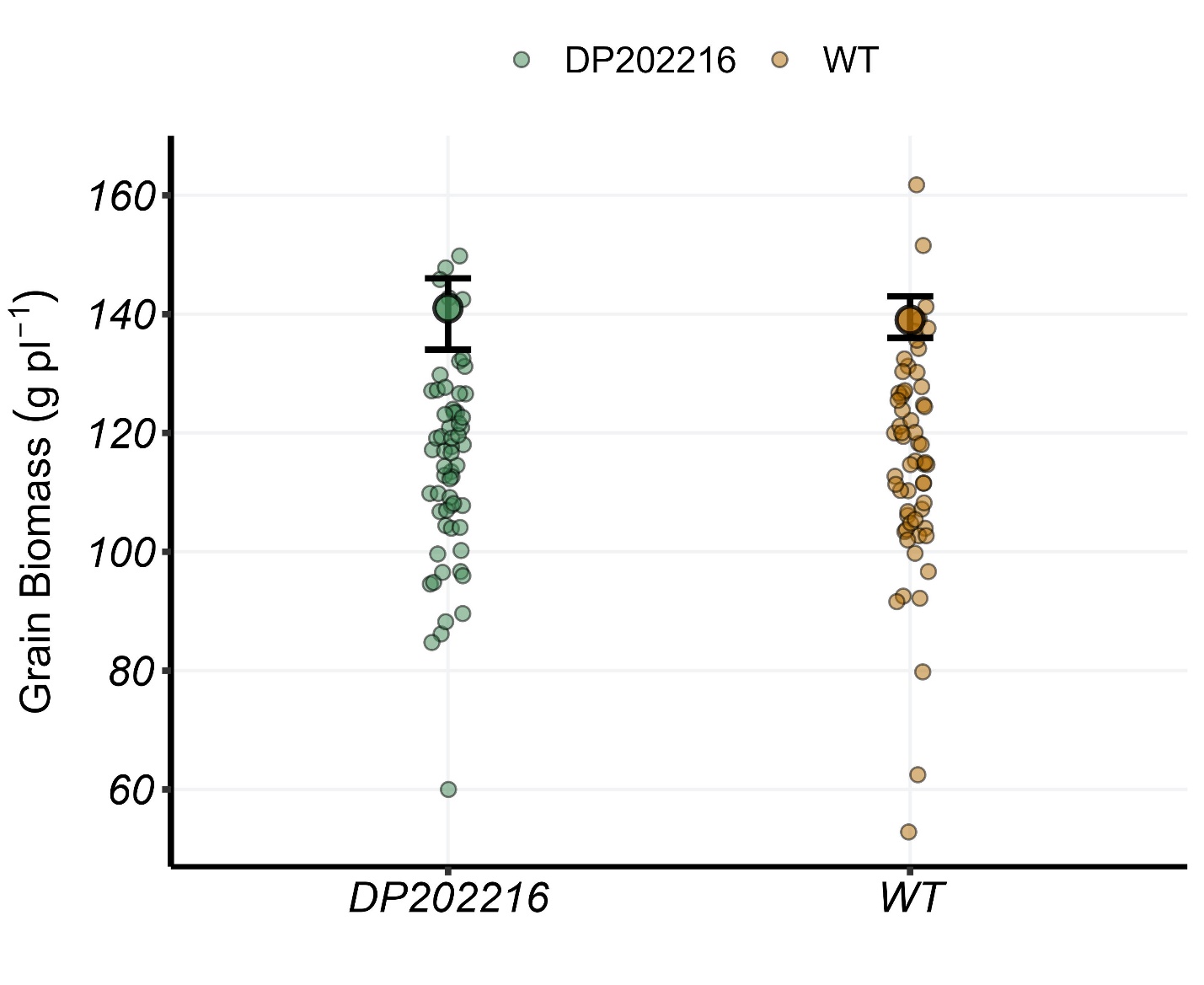
Supplementary Figure 3**. Grain biomass per plant for DP202216 corn hybrids and their respective wild-type (WT). The bigger circles represent the expected value of the grain biomass at the 0.95 quantile obtained from the posterior distribution of the model parameter (intercept only models), while the whiskers represent the 95% credible interval from the posterior distribution. The estimated model parameters are: $Grain Biomass_{i} (g pl^{-1})=139$, for the WT control, and $Grain Biomass_{i} (g pl^{-1})=141$ for DP202216.

**
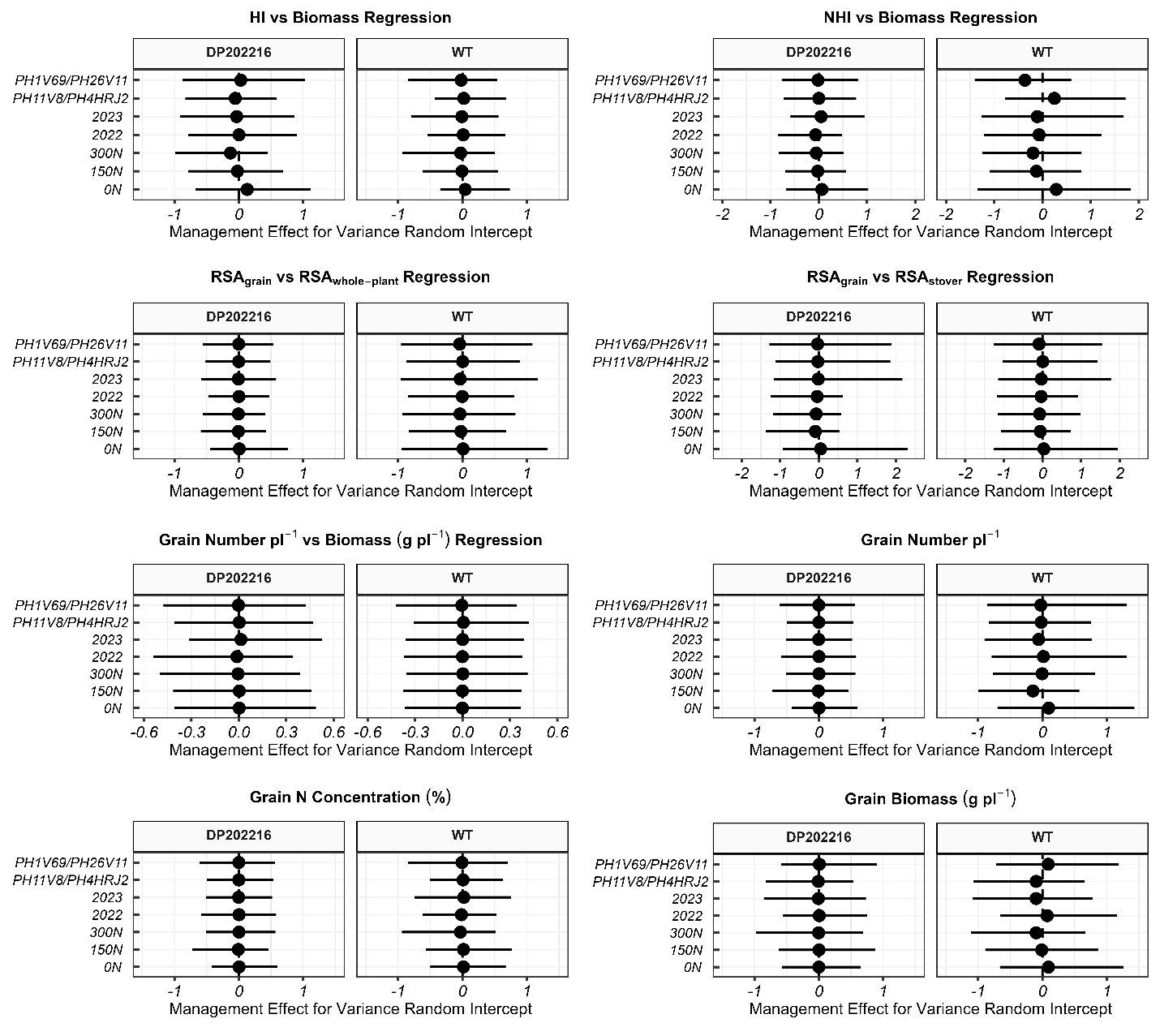
Supplementary Figure 4**. Summary of the nitrogen rate, year of experiment, and hybrid effects across DP202216 event and WT controls. Points and whiskers represent the medians and 95% credible intervals obtained from the posterior distribution of the parameters.

**References**

Bürkner, P.-C. (2017). brms: An R Package for Bayesian Multilevel Models Using Stan. *Journal of Statistical Software* 80, 1–28. doi: 10.18637/jss.v080.i01

Gallais, A., Coque, M., Quilléré, I., Prioul, J., and Hirel, B. (2006). Modelling postsilking nitrogen fluxes in maize ( *Zea mays* ) using ^15^ N‐labelling field experiments. *New Phytologist* 172, 696–707. doi: 10.1111/j.1469-8137.2006.01890.x

Gelman, A., and Rubin, D. B. (1992). Inference from Iterative Simulation Using Multiple Sequences. *Statistical Science* 7, 457–472. doi: 10.1214/ss/1177011136

Posit team (2023). RStudio: Integrated Development Environment for R. Available at: http://www.posit.co/ (Accessed July 4, 2023).

R Core Team (2020). R: A language and environment for statistical computing. Available at: https://www.r-project.org/ (Accessed July 4, 2023).

Ritchie, S. W., and Hanway, J. J. (1982). How a Corn Plant Develops. Iowa State University of Science and Technology, Cooperative Extension Service.
